# An fMRI Study on the Role of Serotonin in Reactive Aggression

**DOI:** 10.1371/journal.pone.0027668

**Published:** 2011-11-16

**Authors:** Ulrike M. Krämer, Jordi Riba, Sylvia Richter, Thomas F. Münte

**Affiliations:** 1 Department of Neurology, University of Lübeck, Lübeck, Germany; 2 Departament de Farmacologia i Terapéutica, Universitat Autònoma de Barcelona, Barcelona, Spain; 3 Helmholtz Center for Neurodegenerative Diseases, Magdeburg, Germany; Cuban Neuroscience Center, Cuba

## Abstract

Reactive aggression after interpersonal provocation is a common behavior in humans. Little is known, however, about brain regions and neurotransmitters critical for the decision-making and affective processes involved in aggressive interactions. With the present fMRI study, we wanted to examine the role of serotonin in reactive aggression by means of an acute tryptophan depletion (ATD). Participants performed in a competitive reaction time task (Taylor Aggression Paradigm, TAP) which entitled the winner to punish the loser. The TAP seeks to elicit aggression by provocation. The study followed a double-blind between-subject design including only male participants. Behavioral data showed an aggression diminishing effect of ATD in low trait-aggressive participants, whereas no ATD effect was detected in high trait-aggressive participants. ATD also led to reduced insula activity during the decision phase, independently of the level of provocation. Whereas previous reports have suggested an inverse relationship between serotonin level and aggressive behavior with low levels of serotonin leading to higher aggression and vice versa, such a simple relationship is inconsistent with the current data.

## Introduction

Aggressive behavior is prevalent in social interactions in both humans and animals. It is highly heterogeneous in its origins and manifestations ranging from verbal insults to full-blown physical violence. Studying its neural basis in terms of critical brain regions and neurotransmitters will provide a better understanding of its conditions and regulating mechanisms. Substantial research has implicated serotonergic functioning in aggressive social interactions, both in animals and in humans [1], [2], [3], [4]. Most studies point to an inverse relationship between serotonin (5-HT) and particularly unrestrained, impulsive aggression in several species [5], [6], [7], [8], [9], although the picture turns out to be less clear for humans [1], [10], [11]. There is still an ongoing debate about the underlying mechanism by which serotonin exerts an impact on reactive aggression. The present fMRI study investigated the role of serotonin in reactive aggression by means of an acute tryptophan depletion and the Taylor Aggression Paradigm [TAP; 12].

Evidence for a regulating impact of serotonin on offensive behavior stems from several lines of research with rodents, monkeys and humans [1]. Correlational and pharmacological challenge studies in humans, for instance, found an inverse relationship between 5-HT functioning and aggression, at least in “extreme” groups, such as criminal offenders [1], [3], [4], [11]. One method to directly lower central 5-HT level and to thereby examine the causal effect of serotonin is acute tryptophan depletion (ATD). A reduced supply of tryptophan, the amino acid precursor of serotonin, leads to lowered cerebral levels of serotonin [13], [14], [15]. A negative correlation between serotonin and aggression has been observed in studies examining ATD effects on laboratory-induced aggression [16], [17]. Interestingly, however, several studies reported an increase of aggression in high trait aggressive subjects only, whereas low-trait aggressive subjects showed even less aggressive behavior after ATD [16], [18], [19], [20]. Cleare and Bond, for instance, reported an increase of aggressive behavior in the Taylor Aggression Paradigm in high trait aggressive subjects only (assessed with the Buss-Durkee Hostility Inventory), while low-trait aggressive subjects showed in fact less aggressive behavior after ATD [16]. This converges with studies using the Point Subtraction Aggression Paradigm [21]: Subjects with high trait aggressiveness or a history of aggression showed increased levels of aggressive responses after ATD, whereas no pharmacological effects or even contrary effects were seen in low aggressive participants [18], [19], [20].

Different suggestions have been made regarding the underlying cognitive and motivational processes that might be affected by tryptophan depletion, and thereby causing increased aggression. Literature related to aggression has mainly focused on the role of serotonin in impulsivity, and explained the effects of ATD with an impairment of inhibitory functions [16], [22], [23]. Other studies have implicated serotonin in stimulus-reward learning and decision-making and suggested that ATD affects the processing of the motivational properties of stimuli [24], [25], [26], [27]. For instance, ATD was found to increase rejection rates of unfair offers in the Ultimatum Game, supposedly resulting from the effect serotonin has on emotional reactions to social feedback [28].

In terms of critical brain regions for aggressive behavior and its regulation, research with psychiatric and neurological patients has highlighted the prefrontal and especially the orbitofrontal cortex [29], [30]. Imaging studies on laboratory-induced reactive aggression in healthy participants [31] identified a network of cortical and subcortical regions related to aggressive behaviour. Lotze et al. [32], presenting in their study one opponent who turned from nice to unfair, could dissociate different roles of the ventral and dorsal prefrontal cortex, with the former related to affective processes and the latter supporting cognitive processes engaged by the social interaction. In another study, Krämer et al. [31] introduced two opponents – one highly and one less provoking – and could thereby disentangle unspecific social interaction processes and cognitive and motivational processes specific to the aggressive interaction. These regions encompassed the dorsal and rostral parts of the ACC, the dorsal striatum as well as the anterior insula which were involved in decision-making and evaluation of the opponent when being provoked.

With the present study, we wanted to investigate the effect of ATD on reactive aggression and its neural correlates. Behaviorally, we expected participants to show more aggressive behavior after ATD. We assessed participants' trait aggressiveness to examine a possible moderating effect on the serotonin – aggression relationship as suggested by previous data [16], [18], [19]. Previous research reported increased ACC and insula activations in response to provocation and related to aggressive retaliation [31]. Moreover, serotonin was shown to change emotional reactions to social feedback and modulate prefrontal and insula activity [28], [33], [34]. Based on these studies, we also expected increased activity especially in the ACC and anterior insula after tryptophan depletion.

## Methods

### Participants

Thirty-six right-handed, male volunteers participated in this study (mean age = 24.8 years,±3.1). No women were included in the study because of changes in serotonergic levels during the menstrual cycle [35]. One participant could not drink the amino acid drink because of nausea, two were removed from further analysis because of technical problems during scanning, two because of extensive movements during scanning and one because he was deemed not to have been completely deceived (as assessed through post-experimental questioning). Thus data of 30 subjects (15 subjects per group; mean age tryptophan depleted group: 24.8, non-depleted group: 24.7) were included in the analysis. The study was performed in agreement with the Declaration of Helsinki and had been approved by the ethics committee of the University of Magdeburg (the affiliation of all authors at the time of the experiment). All subjects gave written informed consent and were paid for participation.

### Procedure

Participants arrived five hours prior to scanning, after an overnight fast from midnight and without alcohol consumption the preceding day. Before administration of the amino acid mixture they filled out the psychological rating and bodily symptom scales (see below). They drank either a balanced amino acid drink containing tryptophan (referred to as *BAL*) or the same mixture without tryptophan (subsequently referred to as *TRP-*). The drinks were assigned in a random double-blind order. We decided to use a between-subject design for the present experiment, as participants can be expected to get suspicious after doing the Taylor Aggression Paradigm once. A repetition of the experiment, as required for a within-subject design, is thus problematic.

The 100g amino acid mixture contained 15 amino acids in proportion identical to human breast milk, except for the omission of glutamic and aspartic acids due to toxicity. The drink consisted of L-Alanin (5.5 g), L-Arginin (4.9 g), L-Cystein (2.7 g), Glycin (3.2 g), L-Histidin (3.2 g), L-Isoleucin (8.0 g), L-Leucin (13.5 g), L-Lysin Monohydrochlorid (8.9 g), L-Methionin (3.0 g), L-Phenylalanin (5.7 g), L-Prolin (12.2 g), L-Serin (6.9 g), L-Threonin (6.5 g), L-Tyrosin (6.9 g) and L-Valin (8.9 g). The balanced drink contained additionally 2.3 g tryptophan. The amino acids were mixed with cold water, sugar and mint and consumed within 10 minutes. The participants received sugar-free chewing gum and water to remove the unpleasant taste. Shortly before the scanning, the psychological rating and bodily symptom scales were administered again.

Before scanning, participants' pain threshold for the thermal stimulation was assessed. Thermal stimuli were delivered by a thermode (3×3 cm thermo-conducting surface; TSA II, MEDOC Inc., Israel) at the back of the left hand. The scanning started approximately five hours after amino acid administration, when the effects of tryptophan depletion are known to be maximal [36], [37]. After scanning, participants filled out a post-experimental questionnaire and rating scales (see below). All participants were given a snack after the testing to reverse any persistent effects of the depletion.

### Questionnaires and rating scales

The participants were assessed with a German inventory for the assessment of factors of aggression (FAF, Fragebogen zur Erfassung von Aggressivitätsfaktoren) [38] and state questionnaires to assess effects of the tryptophan depletion on mood and physical symptoms. With the FAF five sub-scales (spontaneous aggression, reactive aggression, impulsiveness, autoaggression, aggression inhibition) and a control scale (openness) can be obtained. Spontaneous aggression (19 items) refers to unrestrained verbal or physical aggression, items of the reactive aggression scale (13 items) ask for aggressive reactions to some kind of provocation or unfairness, items of the impulsivity scale (13 items) deal with the affective component of aggression. The sum of the scales “spontaneous aggression”, “reactive aggression” and “impulsiveness” gives a reliable measure for outwardly directed aggression (internal consistency Cronbach's alpha = 0.85) and has been proven to differ significantly between either adolescent or adult violent criminals on the one hand and non-violent controls on the other hand [38], providing evidence for its external validity.

Bodily symptoms were assessed with a visual analogue scale (VAS) comprising 8 items (active, passive, calm, agitated, awake, tired, dizzy, nauseous), that had to be rated from “not at all” to “completely”. Aggressive mood was assessed with a VAS [39] that comprised 13 items (angry, quarrelsome, furious, unsociable, aggressive, belligerent, resentful, impatient, hostile, spiteful, annoyed, disgusted and rebellious). This aggression VAS was readministered after the aggression paradigm with the instruction to rate their feelings during the paradigm against the two opponents (separate VAS for each opponent). Additionally, participants answered questions regarding the painfulness of the noxious stimuli and the fairness of the opponents.

### Aggression paradigm

Aggression was elicited and assessed using a modified version of the Taylor Aggression Paradigm [TAP; 12]. Participants were instructed that they were playing successive competitive reaction time trials against one of two opponents outside the scanner taking turns, 24 times against each opponent. The opponents, amateur actors and confederates of the experimenters, met the subject outside of the scanner prior to the experiment to jointly listen to the instructions: They were told that whoever lost would be punished by the opponent with aversive thermal stimulation. The severity of the punishment, that is the temperature of the stimuli, had to be selected for each trial on a scale from 1 to 4. In fact, selections of the putative opponents and outcome of the trials were under control of the experimenter. Participants played 6 times against each opponent in randomized order in each of the four runs. The assignment of the trials to winning or losing was random with the constraint that 50% of the trials were win trials (resulting in 12 win trials against each opponent). At the end of the experiment, subjects were completely debriefed about the deception and the experiment's motivation.

**Figure 1:**
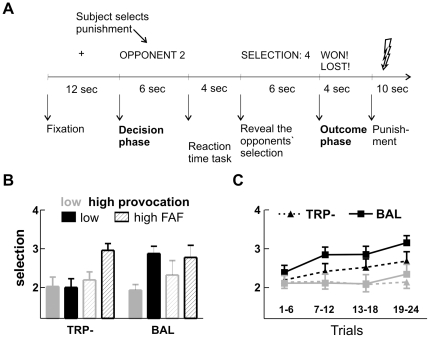
Trial timing and behavioural results. **A** Time course of a single trial under high provocation. The trial began with a 12-s preparation phase. The participant saw the opponent for the upcoming trial and had to select the punishment. After the reaction-time task proper, the participant was informed about the selection of the opponent. Finally feedback was given about the outcome and the participant had to either press a button for the punishment or the temperature of the thermode was increased. **B** Average punishment selections under low (grey) and high (black) provocation separately for low (filled bars) and high (stripes) trait aggressive participants (median split; n = 28). **C** Average punishment selection in low (grey) and high (black) provocation trials, separately for the tryptophan depleted (TRP-) and balanced (BAL) group across the four runs.

At the beginning of each trial, subjects were shown the opponent for the upcoming competition (i.e. “Opponent 1” or “Opponent 2” was displayed on the screen without further information on who of the introduced opponents was “1” or “2”). This was also the prompt to select the magnitude of the punishment (in the following referred to as *decision phase*; duration of 6 sec). The reaction time task proper required the participants to press a button as fast as possible when a picture of a video game character was presented. After that, the selection of the opponent was shown: one opponent selected predominantly lower punishments (mean 1.8; condition of low provocation), the other selected predominantly higher punishments (mean 3.3; condition of high provocation). Finally, feedback was given whether the subject had won or lost (in the following referred to as *outcome phase*; duration of 4 sec). On win trials, they had to elicit the punishment for the opponent by button press, on loss trials they were exposed to the aversive thermal stimulus. The height of the highest possible temperature was individually adapted to the subject's pain threshold. Stimulus presentation and behavioral data acquisition were controlled with Presentation software (www.neurobehavioralsystems.com). The experiment had a duration of 36 minutes, for the timing of each trial see Figure 1A.

The current experimental procedure differed from the previous fMRI study [31] in several points. Instead of the aversive noise, we used thermal stimulation as punishment. As participants are automatically exposed to a high noise level within the MRI scanner, they probably adapt to it to a certain extent. We thus expected the thermal stimulation to be more aversive and to thereby induce more aggressive responses. Moreover, we omitted the “computer” control condition (in which participants were told to outperform their own mean reaction time to avoid punishment) and were thus able to increase the number of trials against human opponents. Finally, we decided to omit the additional punishment for losing in terms of money subtractions to avoid a confound with processes regarding monetary punishment.

### Behavioral analysis

Selections of the participants and their reaction times were scored and compared using repeated measures ANOVA with the within-subject factor Provocation (low and high) and the between-subject factor Tryptophan (TRP; TRP- and BAL). In addition, we analyzed the data of the personality questionnaire (FAF), VAS rating scales and the post-experimental questionnaires to test for effects of the tryptophan depletion and experimental manipulation.

### fMRI acquisition and analysis

We used a 3-Tesla Siemens Magnetom Scanner to collect structural (T1-weighted MPRAGE: 256×256 matrix; FOV = 256 mm; 192 1-mm sagittal slices) and functional images (Gradient-Echo-EPI-sequence; TR = 2000 ms; TE = 30 ms; FOV = 224 mm; flip angle = 80°; matrix = 64×64; slice thickness = 4 mm; four runs of each 270 volumes). 32 transversal slices (3.5×3.5×4 mm voxel) parallel to the anterior commissure-posterior commissure (AC-PC) were obtained. Data analysis included preprocessing (3D motion correction, slice scan time correction and temporal smoothing), spatial smoothing (8 mm full-width at half-maximum Gaussian kernel), co-registration and normalization to Talairach stereotaxic space [40] using Brain Voyager QX.

As in the previous fMRI study [31], the analyses of the functional data were focused on the decision phase and the outcome phase. Random effects analyses were performed on the z-transformed data for the two phases. No provocation effects were seen during the first run as the participants had to first figure out, which opponent was the more aggressive one (see results). We thus did separate analyses of the first run and the second to fourth run. We defined a GLM with predictors for the decision phase under high and low provocation and the outcome phase (winning and losing) for low and high provocation. We included also the reaction time task, the opponent's selection and the punishment phase as predictors of no interest. Additionally, the motion correction parameters were included as covariates.

The fMRI analyses aimed to compare high and low provocation trials during the decision phase as well as during the outcome phase (in win trials only). For both the decision and the outcome phase, we tested for main effects of the provocation as well as for main effects of and interactions with the group factor TRP.

As we did not find a provocation effect in the first run, we additionally asked whether the early neural response to the opponent's behavior predicts the behavioral reactions to the provocation. To this end, we introduced the behavioral provocation effect (effect size, i.e. difference of average selections under high and low provocation relative to the standard deviation) as a covariate in the comparison of high and low provocation trials in the outcome phase of the first run. This allowed us to examine correlations between early provocation effects on the neural level and provocation effects on the behavioral level.

Statistical maps were created using a threshold of p<0.001 (uncorrected for multiple comparisons) with a cluster threshold of 10 voxels. The analysis of correlations between outcome-related activity and the behavioral effect was performed with a more conservative threshold of p<0.0001 (corresponding to an FDR corrected threshold of q<0.02) and an extent threshold of 10 voxels.

## Results

### Questionnaire and rating scales

The mean FAF sum score of aggressiveness was 9.3 (sd = 6.7), and did not differ between the two experimental groups (TRP-: 11.1±7.4; BAL: 7.4±5.5; t_28_ = 1.57, p = .127). The two groups were practically identical regarding their FAF inhibition score (TRP-: 4.4±2.0; BAL: 4.4±1.9).

Participants felt more dizzy and nauseous after the five hours of waiting than at arrival as stated in the bodily symptoms VAS (p<0.05). However, the groups did not differ regarding their bodily symptoms (interaction with TRP: p>0.1). Neither the TRP- nor BAL group showed a change in the aggression VAS when comparing the rating at time 0 and after 5 hours within the subjects and between the groups (all comparisons p>0.1). After the experiment, feelings towards the highly aggressive opponent were consistently rated as more aggressive and negative in all 13 items, most prominently regarding “angry” and least pronounced (but still significant) regarding “impatient” (Wilcoxon signed rank test: all comparisons p<0.05). The two groups did not differ in their rating of the opponents though (all p>0.1).

### Behavioral data

Participants in both groups (TRP- and BAL) selected higher punishments under high than under low provocation (main effect Provocation: F_1,28_ = 14.03, p<0.001), but no significant group differences in the punishment selections were detected (main effect and interaction with TRP: p>0.2). In fact, there was a trend for a smaller behavioral effect in the TRP- group compared to the BAL group. As previous studies reported a diminishing effect of ATD on aggression in low trait aggressive people, we performed exploratory analyses testing for a modulatory role of trait aggressiveness. We did a median split of both groups based on the participants' FAF score (median of the complete sample = 10), yielding a 2×2 factorial design (each group n = 7; excluding one participant in both the TRP- and BAL group to yield equal group sizes). Indeed, low trait aggressive participants in the TRP- group refrained from any aggressive retaliation (effect Provocation: p>0.8), whereas low trait aggressive people in the BAL group reacted aggressively when being provoked (t_6_ = −4.07, p<0.01; interaction TRP×provocation: F_1,12_ = 12.83, p<0.01). No such TRP effect was observed in the high trait aggressive group (F<1), resulting in a significant interaction of TRP×FAF x provocation (F_1,24_ = 5.91, p = 0.023; Figure 1B). As a median split discards information and to verify that this interaction does not depend on the median split, we also performed regression analyses. We tested for correlations between the behavioral provocation effect, i.e. the difference of selections between high and low provocation, and the FAF score separately for TRP- and BAL. As expected from the three-fold interaction in the ANOVA, the regression analysis yielded a significant correlation between personality and provocation effect in the TRP- group (r = 0.57, p = 0.03) but not in the BAL group (r = −0.37, p = 0.18).

When comparing the selections in the four functional runs, both a main effect of Run (F_3,78_ = 5.51, p = 0.003) and an interaction of Run and Provocation were detected (F_3,78_ = 3.77, p = 0.017), reflecting higher selections in later runs, particularly under high provocation (Figure 1C). In fact, selections under high and low provocation did not differ in the first run (p>0.1). These effects were similarly pronounced for the TRP- and BAL groups (interactions of TRP with run: F<1). Further analyses of provocation effects on the neural responses focused accordingly on runs 2–4. Regarding the reaction times of the punishment selections, participants in the BAL group made faster selections under high than under low provocation (low provocation: 1261 ms and high provocation: 1145 ms; t_14_ = −3.78, p = 0.002), whereas reaction times of selections did not differ in the TRP- group (low provocation: 1282 ms and high provocation: 1312 ms; p>0.2). The interaction yielded only marginal significance however (F_1,28_ = 3.29, p = 0.08).

In the post-experimental questionnaire, participants rated the highly aggressive opponent as less fair (Wilcoxon signed rank test: p<0.001) and the highest temperature as more disagreeable than the lowest temperature (Wilcoxon signed rank test: p<0.001). No group differences were observed in these ratings.

### Imaging data

#### Provocation and ATD effects

**Figure 2:**
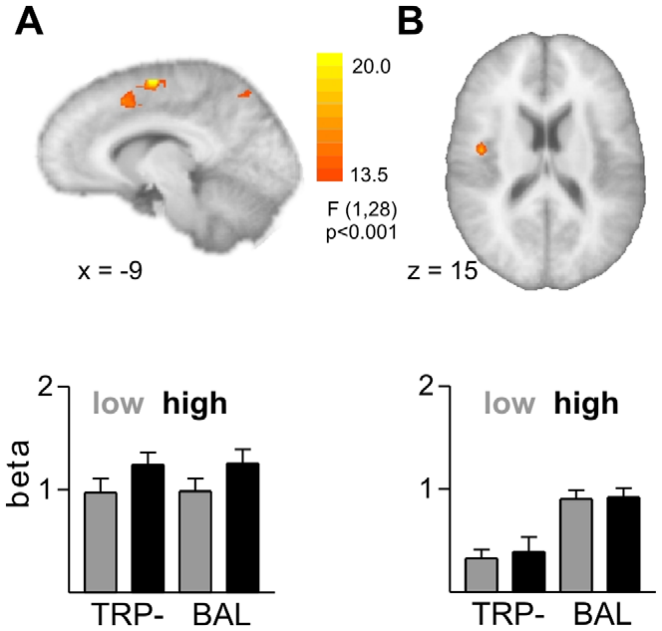
Imaging results for the decision phase. **A** shows the main effect of provocation with an increased BOLD response in the dorsal ACC and below the corresponding beta values separately for the two groups (TRP- left and BAL right). In **B** depicted the main effect of the group factor with a higher BOLD response in the insula for the BAL group. Below the corresponding beta values separately for the two groups (TRP- left and BAL right).

Contrasting high and low provocation trials for the decision phase (runs 2–4) yielded activation in dorsal ACC (BA 24 and 32; Figure 2A), precuneus as well as in premotor and motor cortex (Table 1). In all areas, the BOLD response was stronger for high relative to low provocation. When examining main effects of the tryptophan depletion, we observed increased BOLD responses in the BAL group both in the insula and the cingulate gyrus (Figure 2B; Table 1). Average beta values were extracted from activation clusters for further ROI analyses. We performed post-hoc analyses on the beta values of the insula and cingulate ROI to test for interactions of ATD and trait aggressiveness as in the behavioral data. The ATD effect in the insula was significant in the low trait aggressive participants only (p<0.005), but not in high trait aggressive participants (p>0.1). However, the interaction of ATD and trait aggressiveness yielded marginal significance only (p = 0.081). No significant interactions were detected for the cingulate ROI. No significant interactions of the between-subject factor TRP and the within-subject factor provocation in the whole-brain analyses were observed.

**Table 1:** Brain areas activated during the decision phase.

Region of activation	Laterality	Coordinates	F-value
*Run 2–4: high>low provocation*			
Cingulate gyrus	R	15, −4, 46	21.2
Medial frontal gyrus (BA 6)	L	−9, −7, 58	24.8
Medial frontal gyrus (BA 32)	L	−9, 14, 46	16.6
Precuneus (BA 7)	L	−21, −61, 49	21.2
Precentral gyrus (BA 4)	L	−45, −16, 46	30.2
*Runs 2–4: BAL>ATD*			
Insula (BA 13)	R	42, −7, 16	18.2
Cingulate gyrus (BA 24)	L	−9, 2, 31	21.4
Lingual gyrus (BA 18)	L	−6, −82, −14	18.1

The contrasts of interest for the decision phase yielded several regions defined by strength of effect (p<0.001, uncorrected for multiple comparisons) and size (10 or more voxel). The stereotaxic coordinates of the peak of the activation are given according to Talairach space, together with the F-value for the cluster peak.

Regarding the outcome phase, no main effects or interactions were observed (runs 2–4) when contrasting high and low provocation for win trials.

#### Early outcome evaluation and behavioral provocation effect

**Figure 3:**
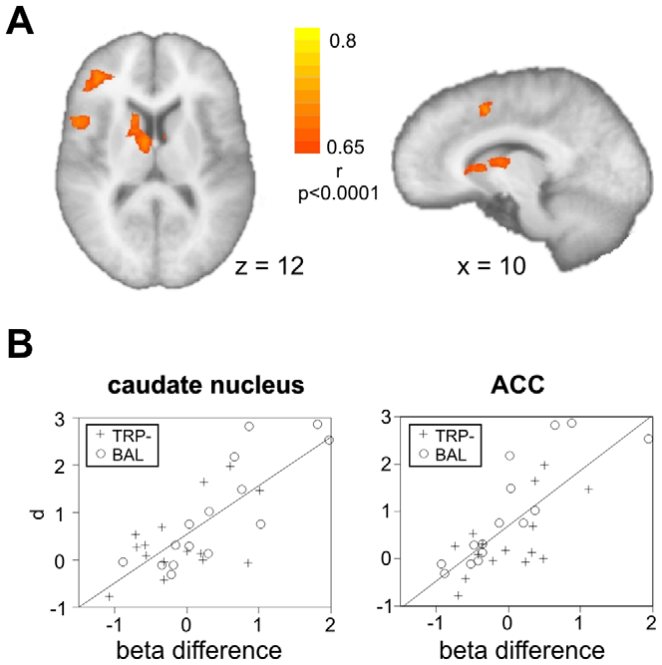
Imaging results for the outcome phase (first run). **A** depicts the results of the correlational analysis for the outcome phase during the first run. Participants with a higher behavioral provocation effect showed an increased neural provocation effect in the caudate nucleus, dorsal ACC, insula and right inferior frontal gyrus. **B** For visualization purpose only, the correlation of the average difference in beta values in the caudate nucleus (left) and ACC (right) with the behavioral provocation effect (effect size d, selection high – low provocation, relative to the pooled standard deviance) is shown with the best linear fit. Participants of the TRP- group are indicated with a cross; BAL participants are shown with a circle.

We did not detect provocation effects during the outcome phase in the runs 2–4, possibly because learning about the opponents' behavior might happen in the beginning mainly, when the feedback is most informative. We therefore asked whether the early neural response during the outcome phase in the first run was correlated with the participants' behavioral response to the provocation. We introduced the behavioral provocation effect as covariate to the GLM contrasting high and low provocation for the outcome phase in the first run (both win and loss trials). The behavioral provocation effect was defined as the effect size of the provocation, i.e. the difference between average selection under high vs. low provocation relative to the pooled standard deviation. Participants' behavioral response to provocation correlated with the neural provocation effect in the right caudate nucleus, right inferior frontal gyrus (BA 47), insula (BA 13) and anterior cingulate cortex (BA 32; Figure 3A and Table 2). No negative correlations were observed. Correlations for the caudate nucleus and ACC are also depicted in Figure 3B.

**Table 2:** Brain regions correlating with behavioral provocation effect.

Region of activation	Laterality	Coordinates	r-value
*Run 1: high>low provocation (outcome)*			
Insula	R	45, 11, 16	0.76
Middle frontal gyrus (BA 10)	R	36, 41, 16	0.77
Inferior frontal gyrus (BA 47)	R	27, 23, −11	0.74
Caudate body	R	12, 14, 7	0.75
Medial frontal gyrus (BA 32)	R	6, 11, 46	0.77

The covariate analysis regarding the outcome phase of the first run and correlations with the behavioral provocation effect yielded several regions defined by correlation (p<0.0001, uncorrected for multiple comparisons; FDR q<0.02) and size (10 or more voxel). The stereotaxic coordinates of the peak of the activation are given according to Talairach space together with the r-value for the cluster peak.

As at least low trait aggressive participants showed no behavioral provocation effect after tryptophan depletion, one might ask, whether the neural provocation effect in this network also depends on trait aggressiveness and ATD. A repeated measures ANOVA on average beta-values in the above-mentioned activation clusters (IFG, insula, caudate nucleus, MFG) with the between-subject factors trait aggressiveness and ATD and the within-subject factors provocation (high vs. low) and outcome (won vs. lost) did not yield significant effects of ATD or trait aggressiveness however.

## Discussion

We examined the effects of tryptophan depletion on reactive aggression and its neural correlates and detected only subtle behavioral and neural effects of lowered serotonin. Importantly, behavioral effects suggested a moderating influence of participants' trait aggressiveness. In addition, we could identify a network of prefrontal (IFG, MFG) and subcortical (caudate) brain regions, whose differential response to the opponent's provocation in the first trials correlated with the participants' aggressive response during the experiment. This was independent of the tryptophan depletion, however.

We detected no clear effect of ATD on aggressive mood or laboratory-induced aggressive behavior in our sample. However, post-hoc analyses revealed reduced reactive aggression in low trait aggressive participants following the ATD. This is in contrast to our hypothesis, but awaits further replication as the sample size was quite small to examine trait-related effects. Note that there was a non-significant tendency for higher aggressiveness scores in the TRP- group. As higher trait aggressiveness has been shown to result in more aggressive behavior in the TAP [41], this tendency might have counteracted the diminishing effect of ATD on aggressive behavior, resulting in a non-significant effect of ATD in the whole sample. Our results go in line with previous studies demonstrating differential effects of ATD on aggressive behavior in low and high trait aggressive people [18], [19], [20]. As outlined in the introduction, previous research featuring the Taylor Aggression Paradigm or the Point Subtraction Aggression Paradigm reported an increase of aggression in high trait aggressive subjects only, while low-trait aggressive subjects showed in fact less aggressive behavior after ATD [16], [18], [19], [20].

This differential effect could result from pre-existing differences in the serotonergic system associated with trait aggressiveness. Evidence for differences in serotonin levels related to trait aggressiveness comes from correlational and pharmacological challenge studies both with healthy people and patients with antisocial personality disorder [1], [3], [4], [11], [42]. Hennig and co-authors, for instance, could present evidence for trait related differential responsiveness of the serotonergic system even within the normal range of aggressiveness [42]. More specifically, individuals scoring high on aggressive hostility were high responders to the selective serotonin reuptake inhibitor (SSRI) citalopram, which was taken as evidence for an elevated postsynaptic sensitivity resulting from lower 5-HT availability. The same authors also reported an association of aggressive hostility with a polymorphism in the tryptophan hydroxylase gene (TPH A779C), an enzyme important for serotonin synthesis. Future studies could directly examine the interactive effects of pre-existing differences in serotonin levels and ATD on reactive aggression by for instance controlling for the genetic makeup of participants [42]. Alternatively, the interaction of the tryptophan effect with personality could be explained in terms of a dual-mode of self-regulation model such that low levels of serotonin lead to a more impulsive, reactive behavior in contrast to an effortful, controlled behavior. As suggested by Carver and co-authors [43], the two modes interact with one's personality style resulting in more impulsive approach behavior in persons with high reward sensitivity and in immobility and withdrawal in persons with high punishment sensitivity. This could also explain the seemingly paradoxical effect of low serotonin on both aggression and depression.

As in our previous study [31], we observed increased activity in the dorsal ACC during the decision phase in high compared to low provocation trials. This underscores the dorsal ACC's known role in cognitive control and response selection and reflects a greater need for cognitive control when being provoked. This effect was not modulated by the tryptophan depletion, however. The BAL group showed a generally increased BOLD response in the right insula during the decision phase compared to the TRP- group, which did not differ between provocation levels. This result is contrary to our hypothesis and does not support previous reports of an inverse relation between serotonin level and insula activation in emotion processing [34], [44]. These studies reported reduced activity in the insula in response to emotional faces after taking citalopram for 21 days [34] and an increased insula response to emotional words after ATD [44]. The present data suggest that the effect of serotonin on insula activation depends on the situational context and differs between assessing emotion-related stimuli and social interactions. Note, that the insula activation in the present study was more posterior than that observed in the previous study [31] and appears to be related to the participants' affective response to the provocation [45], [46], [47]. The present group differences might reflect a reduced affective response to the provocation after ATD, which could explain the tendency for a reduced behavioral provocation effect. This is supported by the observation of a significant ATD effect in low trait aggressive participants only, i.e. in the group that showed reduced aggression after tryptophan depletion. However, as we used a between-group design, group main effects should be interpreted with caution as their specificity for the experimental task is unclear.

We also identified a prefrontal-subcortical network whose activity during the outcome phase in the first run predicted the participants' behavioral reaction to the provocation. Specifically, participants who showed a stronger provocation effect on the neural level differentiated more between the two opponents on the behavioral level. This network comprised the caudate nucleus, anterior insula, dorsal ACC and right inferior frontal gyrus. The anterior insula showed also a provocation effect during the outcome phase in the previous study [31], supposedly related to the emotional response to the provocative opponent. In the current study, this effect was confined to the first run and participants with a strong behavioral provocation effect. The dorsal ACC, right IFG and caudate nucleus were not differentially activated during the outcome phase in the previous study and thus seem to be more specific for the early learning stage during the social encounter. The present results suggest that the identified prefrontal-subcortical network plays a role in establishing a representation of the provocative opponent that is guiding for the behavioral response to the provocation.

The neural effects of provocation were somewhat weaker compared to our previous study [31]. Although a clear behavioral provocation effect was seen in both studies, participants in the present study became overall more aggressive than those in the previous study. This might have blunted provocation effects seen in the imaging data, as the low provocation condition elicited aggressive responses as well.

Our results can be discussed in the light of a recent neurobiological model of punishment which is based mainly on research on conditioned and instrumental learning and economic decision-making [48]. The authors suggest that complex, context-dependent aversive stimuli (such as they occur in the Taylor Aggression Paradigm) are represented in the anterior insula and ventrolateral PFC. Appetitive representations, related, for instance, to the reward of punishing unfairness, are thought to involve the ventromedial PFC together with the dorsal striatum. Instrumental control based on more complex representations of the context and expected future interactions is instigated by a distributed network of prefrontal areas. The present results together with other recent imaging [31], [32] and electrophysiological studies [41], [49] can help to specify this model with respect to reactive aggression. The anterior insula was found to be sensitive to the level of provocation and to be involved both during the decision-making and evaluation of the opponent. We did not observe activations in the ventromedial PFC in the present study, but Lotze and colleagues found vmPFC activity related to compassion with the suffering opponent rather than with appetitive representations [32]. Cognitive control of aggression was found to engage the dorsal ACC in the present study and the dorsomedial PFC in the study of Lotze et al. [32]. The difference might be related to the exact contrast and differences in the paradigms such as playing against two vs. one opponent. In addition, with electrophysiological studies we could demonstrate that cognitive control processes are engaged not only in response to context variables such as provocation but also depend on the player's proneness to aggression [41], [49]. Finally, the dorsal striatum was found to be involved during the early evaluation of the opponent's behavior and correlated with the behavioral provocation effect.

### Conclusions

The present study does not support previous reports of an inverse relationship between serotonin level and aggressive behavior, as only an aggression diminishing effect of ATD in low trait-aggressive participants could be detected. We could replicate provocation-related effects in cingulate gyrus and could identify a network of prefrontal (IFG, MFG) and subcortical (caudate) brain regions, whose differential response to the opponent's provocation in the first trials correlated with the participants' aggressive response during the experiment. It remains an open question how activity in this network is modulated by serotonin or other neurotransmitters such as dopamine. The present study found only a general modulation of anterior insula activity but no provocation specific effect. Future studies should examine whether these effects depend on pre-existing interindividual differences in the serotonergic system or whether other neuromodulators such as dopamine or vasopressin play a more significant role for activity in this network.

## References

[1] Manuck SB, Kaplan JR, Lotrich FE, Nelson RJ (2006). Brain serotonin and aggressive disposition in humans and nonhuman primates.. Biology of aggression.

[2] Olivier B, van Oorschot R (2005). 5-HT1B receptors and aggression: a review.. European Journal of Pharmacology.

[3] Moore TM, Scarpa A, Raine A (2002). A meta-analysis of serotonin metabolite 5-HIAA and antisocial behavior.. Aggressive Behavior.

[4] Coccaro EF (1989). Central serotonin and impulsive aggression.. British Journal of Psychiatry.

[5] Higley JD, Mehlman PT, Poland RE, Taub DM, Vickers J (1996). CSF testosterone and 5-HIAA correlate with different types of aggressive behaviors.. Biological psychiatry.

[6] Mehlman PT, Higley JD, Faucher I, Lilly AA, Taub DM (1994). Low CSF 5-HIAA concentrations and severe aggression and impaired impulse control in nonhuman primates.. American Journal of Psychiatry.

[7] Saudou F, Amara DA, Dierich A, LeMeur M, Ramboz S (1994). Enhanced aggressive behavior in mice lacking 5-HT1B receptor.. Science.

[8] de Boer SF, Lesourd M, Mocaer E, Koolhaas JM (1999). Selective Antiaggressive Effects of Alnespirone in Resident-Intruder Test Are Mediated via 5-Hydroxytryptamine1A Receptors: A Comparative Pharmacological Study with 8-Hydroxy-2-Dipropylaminotetralin, Ipsapirone, Buspirone, Eltoprazine, and WAY-100635.. Journal of Pharmacology and Experimental Therapeutics.

[9] Fish EW, Faccidomo S, Miczek KA (1999). Aggression heightened by alcohol or social instigation in mice: reduction by the 5-HT1B receptor agonist CP-94,253.. Psychopharmacology.

[10] Coccaro EF, Kavoussi RJ (1997). Fluoxetine and impulsive aggressive behavior in personality-disordered subjects.. Archives of General Psychiatry.

[11] Moss HB, Yao JK, Panzak GL (1990). Serotonergic responsivity and behavioral dimensions in antisocial personality disorder with substance abuse.. Biological Psychiatry.

[12] Taylor SP (1967). Aggressive behavior and physiological arousal as a function of provocation and the tendency to inhibit aggression.. Journal of Personality.

[13] Bell CJ, Hood SD, Nutt DJ (2005). Acute tryptophan depletion. Part II: clinical effects and implications.. Australian and New Zealand Journal of Psychiatry.

[14] Hood SD, Bell CJ, Nutt DJ (2005). Acute tryptophan depletion. Part I: rationale and methodology.. Australian and New Zealand Journal of Psychiatry.

[15] Fusar-Poli P, Allen P, McGuire P, Placentino A, Cortesi M (2006). Neuroimaging and electrophysiological studies of the effects of acute tryptophan depletion: a systematic review of the literature.. Psychopharmacology (Berl).

[16] Cleare AJ, Bond AJ (1995). The effect of tryptophan depletion and enhancement on subjective and behavioural aggression in normal male subjects.. Psychopharmacology (Berl).

[17] Pihl RO, Young SN, Harden P, Plotnick S, Chamberlain B (1995). Acute effect of altered tryptophan levels and alcohol on aggression in normal human males.. Psychopharmacology (Berl).

[18] Bjork JM, Dougherty DM, Moeller FG, Cherek DR, Swann AC (1999). The effects of tryptophan depletion and loading on laboratory aggression in men: time course and a food-restricted control.. Psychopharmacology (Berl).

[19] Bjork JM, Dougherty DM, Moeller FG, Swann AC (2000). Differential behavioral effects of plasma tryptophan depletion and loading in aggressive and nonaggressive men.. Neuropsychopharmacology.

[20] Dougherty DM, Bjork JM, Marsh DM, Moeller FG (1999). Influence of trait hostility on tryptophan depletion-induced laboratory aggression.. Psychiatry Research.

[21] Cherek DR (1981). Effects of smoking different doses of nicotine on human aggressive behavior.. Psychopharmacology (Berl).

[22] LeMarquand DG, Pihl RO, Young SN, Tremblay RE, Seguin JR (1998). Tryptophan depletion, executive functions, and disinhibition in aggressive, adolescent males.. Neuropsychopharmacology.

[23] Wingrove J, Bond AJ, Cleare AJ (1999). Tryptophan enhancement/depletion and reactions to failure on a cooperative computer game.. Neuropsychopharmacology.

[24] Rogers RD, Tunbridge EM, Bhagwagar Z, Drevets WC, Sahakian BJ (2003). Tryptophan depletion alters the decision-making of healthy volunteers through altered processing of reward cues.. Neuropsychopharmacology.

[25] Talbot PS, Watson DR, Barrett SL, Cooper SJ (2006). Rapid tryptophan depletion improves decision-making cognition in healthy humans without affecting reversal learning or set shifting.. Neuropsychopharmacology.

[26] Evers EA, Cools R, Clark L, van der Veen FM, Jolles J (2005). Serotonergic modulation of prefrontal cortex during negative feedback in probabilistic reversal learning.. Neuropsychopharmacology.

[27] Cools R, Blackwell A, Clark L, Menzies L, Cox S (2005). Tryptophan depletion disrupts the motivational guidance of goal-directed behavior as a function of trait impulsivity.. Neuropsychopharmacology.

[28] Crockett MJ, Clark L, Tabibnia G, Lieberman MD, Robbins TW (2008). Serotonin modulates behavioral reactions to unfairness.. Science.

[29] Blair R (2004). The roles of orbital frontal cortex in the modulation of antisocial behavior.. Brain and Cognition.

[30] Anderson SW, Bechara A, Damasio H, Tranel D, Damasio AR (1999). Impairment of social and moral behavior related to early damage in human prefrontal cortex.. Nature Neuroscience.

[31] Krämer UM, Jansma H, Tempelmann C, Münte TF (2007). Tit-for-tat: The neural basis of reactive aggression.. NeuroImage.

[32] Lotze M, Veit R, Anders S, Birbaumer N (2007). Evidence for a different role of the ventral and dorsal medial prefrontal cortex for social reactive aggression: An interactive fMRI study.. Neuroimage.

[33] Crockett MJ (2009). The Neurochemistry of Fairness.. Annals of the New York Academy of Sciences.

[34] Arce E, Simmons AN, Lovero KL, Stein MB, Paulus MP (2008). Escitalopram effects on insula and amygdala BOLD activation during emotional processing.. Psychopharmacology.

[35] Rubinow DR, Schmidt PJ, Roca CA (1998). Estrogen-serotonin interactions: implications for affective regulation.. Biol Psychiatry.

[36] Carpenter LL, Anderson GM, Pelton GH, Gudin JA, Kirwin PD (1998). Tryptophan depletion during continuous CSF sampling in healthy human subjects.. Neuropsychopharmacology.

[37] Williams WA, Shoaf SE, Hommer D, Rawlings R, Linnoila M (1999). Effects of acute tryptophan depletion on plasma and cerebrospinal fluid tryptophan and 5-hydroxyindoleacetic acid in normal volunteers.. Journal of Neurochemistry.

[38] Hampel R, Selg H (1975). Fragebogen zur Erfassung von Aggressivitätsfaktoren..

[39] Bond A, Lader M (1986). A method to elicit aggressive feelings and behaviour via provocation.. Biological Psychology.

[40] Talairach J, Tournoux P (1988). Co-planar Stereotaxic Atlas of the Human Brain: An Approach to Medical Cerebral Imaging..

[41] Krämer UM, Büttner S, Roth G, Münte TF (2008). Trait aggressiveness modulates neurophysiological correlates of laboratory-induced reactive aggression in humans.. Journal of Cognitive Neuroscience.

[42] Hennig J, Reuter M, Netter P, Burk C, Landt O (2005). Two types of aggression are differentially related to serotonergic activity and the A779C TPH polymorphism.. Behavioral Neuroscience.

[43] Carver CS, Johnson SL, Joormann J (2009). Two-Mode Models of Self-Regulation as a Tool for Conceptualizing Effects of the Serotonin System in Normal Behavior and Diverse Disorders.. Current directions in psychological science: a journal of the American Psychological Society.

[44] Roiser JP, Levy J, Fromm SJ, Wang H, Hasler G (2008). The effect of acute tryptophan depletion on the neural correlates of emotional processing in healthy volunteers.. Neuropsychopharmacology: official publication of the American College of Neuropsychopharmacology.

[45] Dougherty DD, Shin LM, Alpert NM, Pitman RK, Orr SP (1999). Anger in healthy men: a PET study using script-driven imagery.. Biological Psychiatry.

[46] Phillips ML, Young AW, Senior C, Brammer M, Andrew C (1997). A specific neural substrate for perceiving facial expressions of disgust.. Nature.

[47] Sanfey AG, Rilling JK, Aronson JA, Nystrom LE, Cohen JD (2003). The neural basis of economic decision-making in the Ultimatum Game.. Science.

[48] Seymour B, Singer T, Dolan R (2007). The neurobiology of punishment.. Nature Reviews Neuroscience.

[49] Krämer UM, Kopyciok RP, Richter S, Münte TF (2009). Oscillatory brain activity related to control mechanisms during laboratory-induced reactive aggression.. Frontiers in Behavioral Neuroscience.

